# Cadmium-Induced Bone Toxicity: Deciphering the Osteoclast–Osteoblast Crosstalk

**DOI:** 10.3390/biology14081051

**Published:** 2025-08-14

**Authors:** Shuangjiang He, Kanglei Zhang

**Affiliations:** 1 College of Veterinary Medicine, Yangzhou University, Yangzhou 225009, China; 2 College of Animal Science and Technology, Xinyang Agriculture and Forestry University, Xinyang 464000, China; 2024200009@xyafu.edu.cn

**Keywords:** cadmium, bone toxicity, osteoclast, osteoblast, bone remodeling, environmental toxicology, animal health

## Abstract

Cadmium (Cd), a pervasive environmental and industrial toxicant, bioaccumulates and exerts severe detrimental effects on skeletal integrity across diverse animal species. Cd-induced bone injury manifests as osteoporosis, osteomalacia, and increased fracture risk, posing significant health and welfare concerns for wildlife and livestock inhabiting contaminated ecosystems. The pathogenesis hinges critically on the disruption of bone remodeling, a tightly regulated process orchestrated by osteoclasts (OCs) responsible for bone resorption and osteoblasts (OBs) responsible for bone formation. This comprehensive review synthesizes the latest mechanistic insights into how Cd disturbs OC and OB function and their intricate crosstalk, leading to net bone loss. Cd directly impairs OB proliferation, differentiation, and mineralization capacity through multiple pathways, including the inhibition of Wnt/β-catenin signaling, induction of oxidative stress and mitochondrial dysfunction, promotion of apoptosis and senescence, and disruption of extracellular matrix protein synthesis. Simultaneously, Cd potently stimulates excessive OC formation and activity. It achieves this by upregulating the RANKL/OPG axis, enhancing reactive oxygen species (ROS) production which activates key OC transcription factors, modulating key signaling pathways, and promoting pro-osteoclastogenic inflammatory cytokine release from bone marrow and immune cells. Critically, Cd disrupts the vital communication between OBs and OCs, perturbing the coupling signals essential for balanced remodeling. Emerging evidence highlights roles for Cd-induced epigenetic modifications and autophagy/mitophagy flux alterations. This narrative review integrates the findings from in vivo animal models and in vitro cellular studies, providing potential therapeutic interventions and mitigation strategies for Cd-induced bone toxicity. Understanding these complex and interacting mechanisms provides a foundation for identifying potential therapeutic targets to mitigate Cd bone toxicity in animals and informs ecological risk assessment and management strategies in contaminated environments.

## Simple Summary

Cadmium, a widespread environmental pollutant, poses a significant threat to bone health in both humans and animals. This review focuses on how cadmium exposure disrupts the critical balance between bone-resorbing cells (osteoclasts) and bone-forming cells (osteoblasts). Specifically, Cd directly impairs osteoblasts, hindering new bone formation, and indirectly promotes excessive bone breakdown by overactivating osteoclasts. It achieves this by disrupting vital cellular signals, inducing harmful oxidative stress and inflammation, altering gene regulation, and impairing essential cell functions. Understanding these specific mechanisms is crucial for developing strategies to protect animals, particularly livestock and wildlife in contaminated areas, from cadmium-induced bone diseases like osteoporosis and osteomalacia. This knowledge also informs human health risk assessments.

## 1. Introduction

Cadmium (Cd) ranks as a major environmental contaminant due to its persistence, bioaccumulation through the food chain, and significant toxicity. Natural sources like volcanic activity and anthropogenic activities, particularly mining, smelting, industrial use (batteries, pigments, plastics), and phosphate fertilizer application, contribute to widespread Cd distribution in soil and water [1,2]. Agricultural plants readily absorb Cd, leading to its accumulation in crops and forage, representing the primary exposure route for herbivorous animals and subsequently for carnivores and omnivores [3]. Cd has an exceptionally long biological half-life (10–30 years in humans, similarly prolonged in many animals), primarily stored in the liver and kidneys but also accumulating significantly in bone tissue [4]. This skeletal sequestration acts as a long-term reservoir, facilitating the chronic low-level exposure of bone cells even after external exposure ceases [5].

Bone is a dynamic organ continuously undergoing remodeling, a process essential for maintaining skeletal strength, mineral homeostasis (particularly calcium and phosphate), and mechanical integrity. Bone remodeling involves the coordinated and sequential actions of bone-resorbing osteoclasts (OCs) derived from hematopoietic monocyte/macrophage lineage and bone-forming osteoblasts (OBs) originating from mesenchymal stem cells (MSCs) [6]. Under physiological conditions, OC and OB activities are tightly coupled, ensuring resorbed bone is replaced by newly formed bone to maintain mass and quality [7]. The disruption of this delicate balance, whether towards excessive resorption or deficient formation, results in pathological bone loss, manifesting as osteoporosis (reduced bone mass and microarchitectural deterioration) or osteomalacia (impaired mineralization of newly formed osteoid) [8].

Epidemiological studies in humans and experimental studies in diverse animal models (rodents, rabbits, fish, birds, primates) have unequivocally linked Cd exposure to increased risks of bone fractures, reduced bone mineral density (BMD), and skeletal deformities [9,10]. Cd-induced bone damage is now recognized as a critical adverse effect driving health risk assessments [11]. While renal tubular damage leading to calcium wasting (Itai-Itai disease) contributes to bone demineralization [12], substantial evidence demonstrates that Cd exerts direct and potent toxic effects on bone cells themselves, independent of renal dysfunction [5,13]. This narrative review focuses specifically on the direct and indirect mechanisms by which Cd disrupts OC and OB function and crosstalk, drawing primarily on research published within the last ten years, to elucidate the cellular and molecular underpinnings of Cd-induced bone injury in animal models. Understanding these mechanisms is paramount for developing protective strategies for animals exposed via environmentally or contaminated feed, with implications for wildlife conservation, livestock productivity, and comparative toxicology relevant to human health.

## 2. Cadmium Exposure and Bone Accumulation in Animals

Cd bioavailability and uptake depend heavily on the exposure route (ingestion, inhalation, dermal), chemical speciation, dietary composition (e.g., zinc, calcium, iron, phytate levels), and species-specific physiology [14,15]. Ruminants grazing on contaminated pastures or fed Cd-supplemented diets absorb Cd from the gastrointestinal tract, though potentially less efficiently than monogastrics such as rats due to their unique rumen environment, and still accumulate significant amounts in their organs and bones over time [16]. Fish in contaminated waterways accumulate Cd both directly from water via their gills and skin and through diet, leading to Cd accumulation in their skeletons [17]. Wildlife species serve as sentinels in polluted ecosystems and often exhibit high bone Cd levels, a direct reflection of environmental contamination [18].

**Figure 1:**
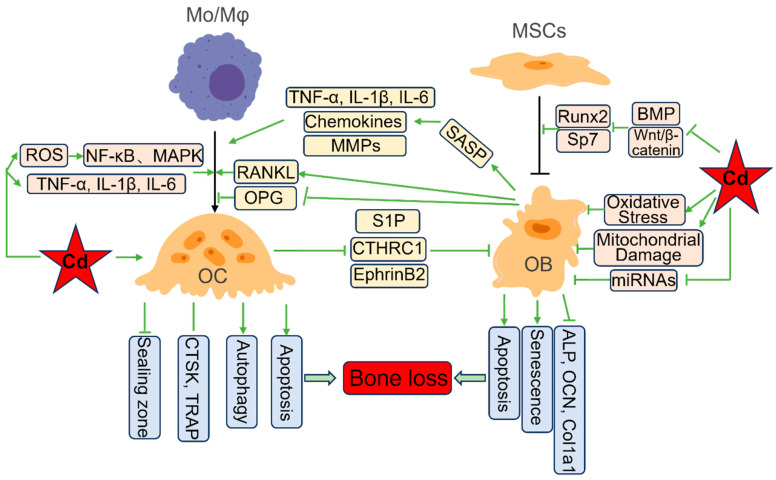
The key mechanisms of cadmium toxicity in osteoclasts and osteoblasts. Cadmium (Cd) promotes osteoclast (OC) differentiation by inducing oxidative stress and pro-inflammatory cytokines (TNF-α, IL-1β, IL-6). Cd also directly disrupts the OC sealing zone or induces OC autophagy and apoptosis. Conversely, Cd inhibits osteoblast (OB) differentiation by downregulating the expression of the key regulators Runx2 and Osterix (Sp7). Cd can directly induce OB oxidative stress and mitochondrial damage, suppress osteogenic miRNAs, and further promote OB apoptosis and senescence. Critically, Cd disrupts OB-OC crosstalk: it inhibits OPG while stimulating RANKL expression in OBs, promoting OC differentiation; Cd-induced senescent OBs secrete pro-inflammatory factors, chemokines, and MMPs, further stimulating osteoclastogenesis; Cd also impairs the release or function of OC-derived coupling factors (S1P, CTHRC1, EphrinB2), inhibiting OB recruitment and bone formation. Collectively, these effects drive net bone loss in vivo.

Once absorbed, Cd is transported in the blood, primarily bound to albumin, metallothionein (MT), or other ligands [19]. Although the liver and kidneys are primary deposition sites, Cd actively accumulates/preferentially deposits in bone, where it incorporates into the hydroxyapatite crystal lattice during bone formation and mineral exchange, substituting for calcium due to its similar ionic radius [20]. Consequently, bone serves as a major long-term storage compartment for Cd. The subsequent release of Cd from bone during physiological resorption processes provides a persistent endogenous source of exposure to bone marrow and bone-lining cells, perpetuating cellular toxicity long after external exposure has ceased [21]. This chronic low-dose exposure within the bone microenvironment is key to understanding the progressive nature of Cd-induced bone damage. The subsequent sections will systematically elucidate the mechanisms by which Cd disrupts bone remodeling, first examining its stimulatory effects on osteoclast formation and function (Section 3); then detailing its inhibitory impacts on osteoblast differentiation, activity, and survival (Section 4); and finally integrating how Cd disrupts the vital crosstalk and coupling between these cell types to drive net bone loss (Section 5). The key mechanisms of Cd toxicity in OCs and OBs are shown in Figure 1.

## 3. Mechanisms of Cadmium Toxicity in Osteoclasts

Osteoclasts (OCs) are multinucleated giant cells, differentiated from mononuclear macrophage (Mo/Mφ) lineage cells, exclusively specialized to degrade the mineralized bone matrix. Their formation (osteoclastogenesis) and activation are primarily governed by the Receptor Activator of Nuclear Factor κB Ligand (RANKL)/Receptor Activator of Nuclear Factor κB (RANK)/Osteoprotegerin (OPG) axis. Macrophage colony-stimulating factor (M-CSF) and RANKL, produced by osteoblasts, osteocytes, and stromal cells, are essential for OC precursor proliferation, differentiation, and fusion [22]. OPG, acting as a soluble decoy receptor for RANKL, inhibits RANKL binding to RANK and thereby suppresses OC formation [23]. Critically, Cd dysregulates this critical axis while simultaneously activating multiple intracellular pathways, ultimately enhancing OC formation and resorptive activity (Figure 1).

### 3.1. Stimulation of Osteoclastogenesis and Resorption

Cd exposure, both in vitro and in vivo, consistently promotes OC formation and bone resorption [24,25,26]. A key mechanism underlying this effect is the dysregulation of the RANKL/RANK/OPG axis. Specifically, Cd increases RANKL expression while decreasing OPG expression in osteoblasts, osteocytes, and stromal cells within the bone marrow microenvironment [27,28], resulting in an elevated RANKL/OPG ratio that provides a potent pro-osteoclastogenic signal. Furthermore, Cd directly enhances the sensitivity of OC precursors to RANKL [29]. Beyond the RANKL/RANK pathway, Cd activates crucial signaling cascades in OCs, downstream of RANK and integrins. These include Nuclear Factor κB (NF-κB), Mitogen-Activated Protein Kinases (MAPKs: p38, JNK, ERK), and Calcium–Calmodulin signaling. The activation of these pathways promotes the induction and activation of Nuclear Factor of Activated T-cells, cytoplasmic 1 (NFATc1), the master regulator of osteoclastogenesis [30,31]. Consequently, Cd exposure upregulates NFATc1 expression and activity in both differentiating and mature OCs [30,32].

### 3.2. Role of Oxidative Stress and Inflammation

Cd is a potent inducer of reactive oxygen species (ROS) generation, primarily through interference with mitochondrial electron transport, the inhibition of antioxidant enzymes (SOD, CAT, GPx), and the depletion of cellular glutathione (GSH) [33,34]. Elevated ROS within OCs and their precursors serves as a critical second messenger. ROS directly activates NF-κB and MAPK pathways, synergizing with RANKL signaling to amplify NFATc1 activation and OC differentiation [35]. Furthermore, Cd induces the production of pro-inflammatory cytokines such as Tumor Necrosis Factor-alpha (TNF-α), Interleukin-1 beta (IL-1β), and Interleukin-6 (IL-6) [36]. These cytokines, particularly TNF-α and IL-1, are potent stimulators of osteoclastogenesis and bone resorption, acting independently or synergistically with RANKL [37]. Cd-induced inflammation thereby creates a paracrine loop that further exacerbates OC activation.

### 3.3. Disruption of Cytoskeleton and Resorptive Function

Mature OCs require a highly organized actin cytoskeleton, forming the characteristic sealing zone, to attach tightly to the bone surface and create the acidic resorption lacuna [38]. Cd exposure disrupts actin ring formation and polarization in OCs. This disruption likely involves Cd interference with calcium signaling, the inhibition of Rho GTPases (key regulators of actin dynamics), and direct oxidative damage to cytoskeletal proteins [39,40,41]. Consequently, Cd impairs the ability of OCs to form functional resorption pits, although Cd exposure stimulates their formation and differentiation. Cd also inhibits the expression and activity of key resorption enzymes, such as Cathepsin K (CTSK) and Tartrate-Resistant Acid Phosphatase (TRAP), potentially through oxidative stress or a direct inhibition pathway [27,42]. Collectively, these effects reveal a complex scenario: Cd promotes the formation of OCs while partially impairing their resorptive machinery; nevertheless, the net effect in vivo is typically increased bone resorption.

### 3.4. Induction of Autophagy and Apoptosis

The effects of Cd on OC survival are concentration- and duration-dependent. Lower, chronic Cd exposure may promote OC survival by inducing autophagy, a cellular recycling process, as a pro-survival mechanism. This can potentially allow OCs to persist longer and contribute to sustained resorption [43]. However, higher or prolonged Cd exposure eventually triggers OC apoptosis (programmed cell death) through mitochondrial dysfunction (cytochrome c release), caspase activation (particularly caspase-3), and ER stress [13]. This delicate balance between autophagy-mediated survival and apoptosis ultimately determines the impact of Cd on OC lifespan and resorptive activity within the bone.

## 4. Mechanisms of Cadmium Toxicity in Osteoblasts

Osteoblasts (OBs), derived from mesenchymal stem cells (MSCs), synthesize the organic bone matrix (osteoid) and facilitate its mineralization. OB differentiation involves the sequential activation of key transcription factors (Runx2, Osterix, ATF4) and signaling pathways [44]. However, Cd exerts multiple direct inhibitory effects on OBs, thereby inhibiting bone formation (Figure 1).

### 4.1. Inhibition of Proliferation, Differentiation, and Mineralization

Cd exposure suppresses the proliferation of both MSCs and pre-osteoblasts [13,27]. Critically, Cd potently inhibits OB differentiation, primarily by downregulating the expression and transcriptional activity of the master regulators Runt-related transcription factor 2 (Runx2) and Osterix (Sp7) [45]. This downregulation is mediated through multiple mechanisms, including the inhibition of bone morphogenetic protein (BMP) signaling and, most significantly, the disruption of the canonical Wnt/β-catenin pathway. Wnt signaling is crucial for OB commitment, differentiation, and survival [46], and Cd suppresses the osteogenesis of BMMSCs via the inhibition of this pathway [47]. Consequently, Cd exposure reduces the expression of key OB markers like Alkaline Phosphatase (ALP), Osteocalcin (OCN), and Collagen type I alpha 1 (Col1a1). Furthermore, Cd severely impairs matrix mineralization, the ultimate step of OB function. This impairment arises from both the inhibition of ALP activity (essential for providing phosphate) and direct interference with hydroxyapatite crystal nucleation and growth, partly through calcium substitution and the disruption of the crystal structure [24,48].

### 4.2. Induction of Oxidative Stress and Mitochondrial Damage

OBs are particularly susceptible to Cd-induced oxidative stress. Cd accumulates in mitochondria, disrupting the electron transport chain (ETC), uncoupling oxidative phosphorylation, and inducing excessive ROS production [49,50]. This mitochondrial damage not only depletes ATP (essential for OB anabolic activities) but also contributes to the depletion of cellular antioxidants like glutathione (GSH), further exacerbating oxidative damage [51]. The resulting ROS directly damages OB cellular components (lipids, proteins, DNA), activates pro-apoptotic or pro-senescent stress kinases (e.g., p38 MAPK), and inhibits key signaling pathways such as Wnt and BMP [52]. Furthermore, Cd disrupts mitochondrial dynamics (fusion/fission) and quality control via mitophagy, leading to the accumulation of damaged mitochondria and sustained ROS generation [53].

### 4.3. Promotion of Apoptosis and Senescence

Cd potently induces OB apoptosis, triggering both the intrinsic (mitochondrial) pathway, involving Bax/Bak activation, cytochrome c release, and caspase-9/-3 activation, and the extrinsic (death receptor) pathway [50]. Additionally, Cd-induced endoplasmic reticulum (ER) stress, resulting from protein misfolding and calcium dyshomeostasis, contributes to OB apoptosis via the PERK-CHOP pathway [54]. Beyond apoptosis, Cd promotes OB senescence, a state of irreversible cell cycle arrest characterized by morphological changes, Senescence-Associated Beta-Galactosidase (SA-β-gal) activity, and the secretion of pro-inflammatory factors known as the Senescence-Associated Secretory Phenotype (SASP) [13,55]. These senescent OBs not only lose their bone-forming capacity but also secrete factors that negatively impact neighboring cells and promote OC activation (see Section 5).

### 4.4. Epigenetic Modifications

Emerging evidence indicates that Cd disrupts the OB epigenome by dysregulating microRNA (miRNA) expression. Specifically, Cd alters key osteogenic miRNAs (e.g., miR-29b, miR-133a, miR-135a, miR-141-3p, miR-205), suppressing OB differentiation and function [56,57]. These epigenetic modifications induce the long-lasting repression of the osteogenic program.

## 5. Disruption of Osteoblast–Osteoclast Crosstalk and Bone Remodeling Coupling

The coordinated action of OBs and OCs relies heavily on intricate bidirectional communication. Cd disrupts this crosstalk at multiple levels, uncoupling bone resorption from formation and tipping the balance towards net bone loss (Figure 1).

### 5.1. Altered RANKL/OPG Axis by Osteoblasts

As mentioned in Section 3.1, Cd exposure dramatically shifts the RANKL/OPG ratio toward RANKL dominance in OBs, osteocytes, and stromal cells [58,59]. This alteration is a key pathway by which Cd indirectly stimulates OC activation. Cd simultaneously suppresses OPG expression and stimulates RANKL expression in these cells, establishing a sustained pro-resorptive signal. This dysregulation is driven by Cd-induced oxidative stress, inflammatory cytokines within the bone microenvironment, and direct effects on OB gene transcription [31].

### 5.2. Senescence-Associated Secretory Phenotype (SASP)

Cd-induced senescent OBs develop a SASP featuring pro-inflammatory cytokines (e.g., IL-6, IL-1α/β, TNF-α), chemokines, matrix metalloproteinases (MMPs), and, notably, elevated RANKL [13]. This SASP generates a local inflammatory milieu that directly stimulates OC formation/activity while suppressing neighboring healthy OB function, thereby amplifying bone loss through paracrine signaling. Consequently, senescent cells serve as persistent paracrine disruptors within the bone remodeling compartment.

### 5.3. Impaired Coupling Factors

Physiologically, coupling factors—such as those released from the resorbed bone matrix (e.g., TGF-β, IGF-1, BMPs) or produced by OCs (e.g., Sphingosine-1-phosphate [S1P], Collagen triple helix repeat containing 1 [CTHRC1], EphrinB2)—stimulate subsequent OB recruitment and bone formation [60]. However, Cd may impair the release or bioavailability of these coupling factors during resorption. More critically, Cd compromises OB responsiveness to anabolic signals. For example, it inhibits TGF-β and BMP signaling pathways in OBs [61,62,63], thereby blunting the pro-osteogenic coupling response.

## 6. Role of Osteocytes and Other Cell Types

Although OBs and OCs are primary targets, Cd toxicity extends to other bone cells. Osteocytes, terminally differentiated OBs embedded within the mineralized matrix, are the most abundant bone cells and serve as key mechanosensors and endocrine regulators of remodeling [64,65]. Cd accumulates in osteocytes and induces their senescence [66]. Dying osteocytes release potent signals that stimulate OC recruitment and resorption [67,68]. Furthermore, Cd impairs bone marrow mesenchymal stem cells (BMSCs), reducing their proliferative capacity and diverting differentiation from the osteoblastic lineage toward adipogenesis [27], thereby depleting OB precursors. Cd also activates bone marrow immune cells (e.g., macrophages), releasing pro-inflammatory cytokines such as TNF-α. TNF-α is a critical regulator of osteoclastogenesis, inducing both the differentiation of osteoclasts and their bone-resorbing activity [69].

## 7. Animal Models and Species Differences

Research on Cd bone toxicity primarily relies on animal models. Rodents (rats, mice) are the most common due to their genetic tractability and relatively short lifespans, facilitating chronic exposure studies [70]. Typical exposure routes include subcutaneous injection, oral gavage, or drinking water supplementation. These models consistently demonstrate Cd-induced reductions in BMD, trabecular bone volume, and bone strength, alongside elevated bone resorption markers and histomorphometric evidence of increased OC surfaces [71]. Emerging fish models (e.g., zebrafish, medaka) enable studies on skeletal developmental toxicity (vertebral deformities) and aquatic ecosystem impacts [72]. In birds (e.g., laying hens), Cd exposure reduces eggshell quality (via disrupted calcium mobilization) and induces skeletal damage [73,74]. Non-human primate studies remain rare but offer high relevance for human risk extrapolation. Notably, susceptibility to Cd varies to some extent among animal species (Table 1), depending on species, age (growing animals and older individuals often more vulnerable), nutritional status (calcium, vitamin D deficiency exacerbates toxicity), and exposure duration/dose [75]. The collective findings from the above animal models have provided a crucial foundation for revealing the mechanism of Cd-induced bone toxicity and for potential therapeutic interventions.

## 8. Potential Therapeutic Interventions and Mitigation Strategies (Research Focus)

Understanding the mechanisms of Cd bone toxicity opens avenues for potential interventions. Current research focuses on several strategies:

Antioxidants: N-Acetylcysteine (NAC), Melatonin, and natural antioxidants (Curcumin, Resveratrol, Quercetin) show promise in mitigating Cd-induced oxidative stress in bone cells and preserving bone mass in animal models [45,76].

Anti-Resorptives: Bisphosphonates (e.g., Zoledronate), which target OCs, reduce Cd-induced bone loss in rodents by inhibiting excessive resorption [77]. Denosumab (anti-RANKL antibody) is a potential candidate but requires specific testing in Cd models [78].

Anti-Inflammatories: Agents targeting TNF-α (e.g., Etanercept) or IL-1 could potentially interrupt the pro-osteoclastogenic inflammatory loop driven by Cd [79,80].

Wnt Pathway Modulators: Inhibiting sclerostin (using anti-sclerostin antibodies like Romosozumab) could counteract Cd-induced Wnt inhibition and stimulate bone formation [81]. Lithium (GSK-3β inhibitor) represents another potential Wnt activator under investigation.

Senolytics: Drugs selectively eliminating senescent cells (e.g., Dasatinib + Quercetin) could remove the source of the detrimental SASP, potentially improving the bone microenvironment following chronic Cd exposure [82].

Nutritional Interventions: The adequate dietary intake of calcium, vitamin D, zinc, and Selenium is crucial for reducing Cd absorption and bioavailability and supporting bone health [83].

Chelation Therapy: While primarily used for acute poisoning, chelators like EDTA or DMPS can reduce body burden; however, their effectiveness against chronic bone Cd accumulation and associated toxicity, along with potential side effects, requires careful evaluation [84].

Importantly, most of these interventions remain at the preclinical (animal model) stage (Table 2), highlighting a significant translational gap.

## 9. Conclusions

Research over the past several decades has significantly advanced our understanding of the intricate mechanisms by which Cd disrupts bone remodeling through direct and indirect toxicity to osteoclasts and osteoblasts. Cd hijacks fundamental cellular processes: it promotes OC formation and activity primarily by dysregulating the RANKL/OPG axis and inducing ROS/inflammation while simultaneously crippling OB differentiation, function, and survival by inhibiting Wnt signaling, inducing oxidative stress, mitochondrial damage, apoptosis, and senescence. Critically, Cd disrupts the vital crosstalk between these cells, uncoupling resorption from formation. The role of osteocytes, SASP, and epigenetics adds further complexity to this pathological network.

## 10. Future Perspectives

Despite these advances, critical knowledge gaps and limitations exist. First, the overwhelming majority of mechanistic evidence derives from in vitro studies and in vivo animal models. Extrapolating these findings to human health, particularly concerning the long-term consequences of low-dose, environmentally relevant Cd exposure initiated early in life on skeletal development, peak bone mass attainment, and fracture risk in adulthood, remains uncertain and requires dedicated human epidemiological and biomarker studies. Second, significant heterogeneity exists across studies in Cd exposure routes (oral gavage, drinking water, injection), doses (often supraphysiological), and durations, complicating comparisons, risk assessment, and establishing clear dose–response relationships. Third, while Cd-induced epigenetic modifications are recognized, the precise contribution of these changes to persistent bone dysfunction after exposure cessation or potential transgenerational effects remains largely unexplored in longitudinal and multigenerational animal studies. Fourth, the role of Cd-induced gut microbiota dysbiosis and its impact on bone health via the gut–bone axis is an emerging area requiring substantial investigation to understand this indirect pathway. Fifth, the development and validation of sensitive and specific biomarkers for the early detection of Cd-induced bone damage in both animals and humans are urgently needed. Furthermore, translating mechanistic insights from animal models into strategies for protecting human bone health and refining regulatory frameworks for environmental Cd levels is paramount.

Addressing Cd contamination requires integrated strategies: reducing environmental emissions, remediating contaminated land, managing Cd levels in fertilizers and feed, and ensuring adequate nutrition for exposed animals and humans. The insights gained from mechanistic studies on OC and OB dysfunction are vital for developing targeted pharmacological or nutritional strategies to protect bone health in animals inhabiting Cd-contaminated environments and for informing human risk assessment. This research not only safeguards animal welfare and productivity but also provides critical data for ecological risk assessment and public health efforts to mitigate the impact of this pervasive toxicant on skeletal health.

## Figures and Tables

**Table 1:** Comparison of Cd-induced bone toxicity across animal species.

Animal Species	Exposure Route(s)	Major Skeletal Effects	Susceptibility Level	References
Rodents (Rats, Mice)	SC injection, Oral gavage, Drinking water	Significant ↓ BMD, ↓ trabecular bone volume, ↓ bone strength, ↑ OC activity	High	[70,71]
Fish (Zebrafish, Medaka)	Water (gills/skin), Dietary	Severe vertebral deformities, spinal curvature, mineralization defects (prominent developmental toxicity)	High	[72]
Birds (Laying hens, Quail)	Dietary	Significant ↓ eggshell quality (disrupted Ca metabolism), ↓ bone mineralization, ↓ bone biomechanical properties	Moderate–High	[73,74]

Note: (↑) increase; (↓) decrease.

**Table 2:** Potential therapeutic strategies targeting Cd bone toxicity mechanisms.

Target/Mechanism	Potential Intervention	Proposed Action	Evidence Level (Animal Models)	References
General Oxidative Stress	N-Acetylcysteine (NAC), Melatonin	↑ GSH synthesis, direct ROS scavenging	Rodents	[45,76]
	Curcumin, Resveratrol, Quercetin	Antioxidant, anti-inflammatory properties	Rodents	[85,86]
Osteoclast Activation	Bisphosphonates (e.g., Zoledronate)	Induce OC apoptosis, inhibit resorption	Rodents	[77]
	Anti-RANKL (e.g., Denosumab analog)	Block RANKL binding to RANK, inhibit OC formation	*Hypothesized*	[78]
Inflammation	TNF-α Inhibitors (e.g., Etanercept analog)	Neutralize TNF-α, reduce pro-osteoclastogenic signaling	Limited Rodent	[80]
	IL-1 Receptor Antagonist	Block IL-1 signaling	*Hypothesized*	[79]
Wnt Inhibition (OBs)	Anti-Sclerostin Antibody	Neutralize SOST, restore Wnt signaling, ↑ OB formation	*Hypothesized* (Romosozumab known effective in osteoporosis)	[81]
	GSK-3β Inhibitors (e.g., Lithium)	Stabilize β-catenin, enhance Wnt signaling	In vitro/*Hypothesized*	[87]
Cellular Senescence	Senolytics (e.g., Dasatinib + Quercetin)	Eliminate senescent cells (OBs, others), remove SASP source	Emerging in other contexts	[82]
Nutritional Support	Adequate Calcium and Vitamin D	↓ Cd absorption, support mineralization, bone health	Rodents/Livestock	[83]
Cd Burden Reduction	Chelators (e.g., EDTA, DMPS—cautious use)	Bind Cd, enhance excretion (primarily acute/high dose)	Variable efficacy, side effects	[84]

Note: ↑ upregulate; ↓ downregulate.

## Data Availability

Not applicable.
